# The Origins of Food Supplied to an Australian Public Hospital

**DOI:** 10.3389/fnut.2022.771742

**Published:** 2022-03-15

**Authors:** Kathy Faulkner, Heather Gilbertson, Judi Porter, Jorja Collins

**Affiliations:** ^1^Department of Nutrition, Dietetics and Food, Monash University, Melbourne, VIC, Australia; ^2^Department of Nutrition and Food Services, Royal Children’s Hospital, Melbourne, VIC, Australia; ^3^Institute for Physical Activity and Nutrition, School of Exercise and Nutrition Sciences, Deakin University, Geelong, VIC, Australia

**Keywords:** institutional foodservice, local food procurement, food origin, food supply, hospital, healthcare

## Abstract

Local food procurement by public institutions such as hospitals offers multiple benefits including stimulating the local economy, creating jobs, and building resilience within the food supply. Yet no published study has attempted to quantify the local food purchase by hospitals. This baseline is needed to identify gaps, set targets, and monitor change. The objective of this study was to investigate the origin of food supplied to a metro tertiary public hospital and to describe the proportion of food budget spent on items with ingredients grown in Australia and “locally” within the state of Victoria. Food procurement data were collected and analyzed during October 2020-April 2021. All items purchased by the cook fresh kitchen supplying meals to inpatients and two childcare centres during an 8-day menu cycle period were audited. Following an inspection of food packaging labels to determine country of origin, data on the proportion of Victorian content were collected from manufacturers and suppliers of foods containing Australian ingredient. Almost 80% of the food budget (AU $17,748 and 200 items) was spent on items containing significant (at least 75%) Australian content, while 11% was spent on entirely imported foods. The specific geographic origins of 55% of the budget spent on “Australian” food remain unknown as information from manufacturers and suppliers was not available. Where data were available, 3% of food budget was attributed to entirely Victorian grown foods, including fresh fruit, vegetables, and poultry. A considerable proportion of Australian grown foods are purchased by this hospital, but it is largely unknown whether these are local, from the state of Victoria, or not. Tracing and sharing of food origin data, a clear definition for “local” food, and an understanding of Victorian food growing industries are needed to progress the “local food to hospital” agenda.

## Introduction

Global industrialized food systems are productive, but they are wasteful and unsustainable. They contribute to injustices in the distribution of the food supply world-wide (1, 2). They have evolved because of artificially cheap fossil fuels, technological advances, and world trade agreements (3). Institutional foodservice, with its considerable scale and yet limited individual resources, both contributes to and is generally reliant upon this dominant global food supply (4).

But global food systems are in crisis. Their vulnerabilities, exposed by the COVID-19 pandemic, highlight a reliance on international transport systems, the disruption of which can impact any country (5, 6) even a net food exporting country like Australia (7, 8). It is these threats to food security that have seen increased interest in local food by consumers, local government, community organizations and the not-for-profit sector (5, 8).

Local food systems are seen to offer multiple benefits from improving the quality (freshness), safety and seasonality of the food supply, to stimulating a local agriculture sector, creating employment opportunities, and conserving culture and identity (3). They can also assist in establishing shorter supply chains, connecting farmer with consumer, and creating resilience within the broader food supply (3). Yet “local food” is not a well-defined concept; its meaning varies according to different individual values and perspectives (9). The basic premise, however, is that local food is differentiated by place; it is grown or produced within a geographic region near to where it is consumed. This geographic distance can vary substantially, and one survey found consumers perceived it to include everything from “the closest relevant producer” to “within a radius of 640 km” (3).

It must acknowledged that a debate exists as to the benefits and disadvantages of the local food approach. Critics provide the argument against localism as an all-encompassing solution to the environmental, social and health impacts of the current “global-agro” food system (10, 11). They do not entirely dismiss the concept but rather, they herald a warning to policy makers, that while the local food approach is politically appeasing and caters to a wide range of values, without clear definition or limits, it can perpetuate the same problems caused by the industrialized global food system (11). An article by Sumner et al. (12) provides an example of local food procurement that incorporates values of community connection and culture. The authors argue that it is the incorporation of these values that allow the program to successfully address social, environmental and health risks.

Use of public procurement by governments as a driver of market forces is not new. Institutional foodservice, with its considerable buying potential, offers governments the opportunity to support local food systems by establishing a “local-food-to-institution” agenda. The food budget of healthcare is not insignificant, worth more than half a billion Australian dollars (13). If redirected to into local food procurement the opportunities for regional development and planetary health benefits would be considerable.

Foodservice is broadly defined as establishments which provide food and meals prepared outside the home (14). In commercial foodservice selling food is a core business, e.g., restaurants, but in institutional foodservice, food and meals are provided as part of a wider service. It includes (but is not limited to) private and public hospitals, aged care facilities, correctional facilities, the education sector, Defense and workplace corporate canteens (14).

Successful “local-food-to-institution” has been established around the world. Within Australia, there is a growing public awareness of the need to support local food systems, and in response to this sentiment, some local and state governments, universities, and charity organizations are advocating for reform (15–17). But Australia has been slow to engage in the “local-food-to-institution” movement. This may be due to, to limited government policy support or lack of opportunity. Unlike most of Europe and Northern America, Australia does not offer a government funded school lunch program and residential dining halls in universities and cafeterias in workplaces are less common. Indeed, the institutional foodservice market accounted for just 13% (AU $7.4 billion) of the annual foodservice turnover in the 2019–2020 financial year (18), compared with the United States, in which the institutional foodservice market accounted for more than 27% (US $200 billion) of foodservice sales in 2012 (14).

Hospitals have a consistent year-round demand for a sizeable amount of food, making them an ideal institutional setting for local food procurement. The review by Carino et al. (19) comprehensively described the environmental sustainability literature on hospital foodservice and noted that strategies to achieve sustainable food procurement, such as local food, was the second most explored issue, indicating interest in understanding its enablers and impacts. Realizing this requires moving away from the traditional procurement model in healthcare of using group purchasing organizations (GPO). Indeed there are a number of studies, that have identified GPOs as a barrier to the “local-food-to-institution” agenda (19–21). Another study by Carino et al. (22) found that hospital staff perceived procurement to be restricted by current supply contracts. But there are a number of barriers to introducing a local-food-to-hospital program that GPOs have been designed to overcome. GPOs unite hospitals to establish collective buying power and negotiate contracts based on best-dollar value terms to provide cost savings. Contracts are negotiated to be responsive to different foodservice production models, demand for ingredient consistency and volume, food safety and streamlined procurement and delivery (19, 20, 23). Nevertheless, farm-to-hospital procurement programs supported by *Health Care Without Harm* have been achieved in the United States by setting clearly defined targets and definitions, facilitating hospital connections with local farmers and food hubs, providing staff education and resources, auditing hospital compliance and celebrating achievements (21). In the United Kingdom, local food procurement has been identified by the National Health Service, as one strategy it will employ in its efforts to achieve net zero emissions by 2040 (24).

Healthcare in Australia is predicted to grow due to an aging population, presenting an opportunity to capitalize on its purchasing power to positively impact food systems and supply chains (18). Indeed, the Victorian state government has committed to the provision of local foods on public hospital menus (25). While other state governments have committed to the principle of “buying local” and in fact Queensland has released a guide and supplier directory to support government officials in this endeavor (26), no other state has explicitly identified healthcare food procurement as a means of supporting local.

However, it is currently unknown how much food is locally sourced (and how much is not) by any Australian hospital. A recent review by Carino et al. (19) sought to evaluate a local food-to-hospital program or toolkit, none was found to have quantified the local-food-to-hospital procurement prior to implementing a program. Furthermore, the authors are unaware of any published research, to date, that has attempted to quantify local food procurement in hospitals. Without this baseline data about existing patterns of food procurement, gaps cannot be identified, improvements over time cannot be monitored and comparisons cannot be made. This research is a critical starting point to evaluate implementation of this State government policy (25).

Therefore, the aim of this study is to investigate the origin of food supplied to a metro tertiary public hospital and to describe the proportion of food budget spent on items with ingredient grown in Australia and also “locally” within the state of Victoria.

## Materials and Methods

This was a cross sectional study involving the collection and analysis of food procurement data in a Victorian metropolitan hospital. A two-step audit process was followed, informed by a process guide developed by My Sustainable Canada and Canadian Coalition for Green Health Care (22). Phase 1 (October 2020) involved collecting and identifying the country of origin of all foods purchased and Phase 2 involved determining local content. Local food was defined as any food containing ingredients grown (in the case of produce) or raised (in the case of livestock) within Victoria, the state where the hospital is located.

Approval for this project was granted by the Royal Children’s Hospital (Melbourne) Research Office (HREC Reference Number: QA/68712/RCHM-2020). Verbal consent was sought from all participants.

### Setting

The study was conducted at a 350-bed metro-tertiary children’s hospital in Victoria, Australia. The hospital is funded under a Public Private Partnership arrangement, whereby all the clinical and support services (including food services) are publicly funded by the State Government, while the private sector is responsible for maintaining the hospital building and infrastructure (27).

The hospital foodservice uses a cook fresh model where meals are prepared and cooked for service in an onsite kitchen. All food is sourced and purchased according to the GPO for participating health services in Victoria. Food for the hospital is purchased from both broadline distributors who carry a large range of predominately processed and packaged foods and from smaller suppliers of predominately fresh and perishable foods (e.g., meat, bread, fruit, vegetables, and dairy). The foodservice operates on an 8-day cyclical menu and includes 3 meals and 1 snack per day with ward pantry provisions providing for additional snacks during the day for patients and breast-feeding mothers (approximately 200 meals per service). The foodservice also provides meals to both an on-site and neighboring child-care facility (approximately 160 meals every weekday).

### Data Collection

The proportion of Australian and local ingredient content was determined for all food items purchased over all days of the 8-day cycle menu. Data were expressed as a percentage of the food budget as recommended in the Canadian audit tool (28) used for this study. It provides a better comparator than number of “items” and it overcomes the issue of volume (L) versus mass (kg).

**FIGURE 1:**
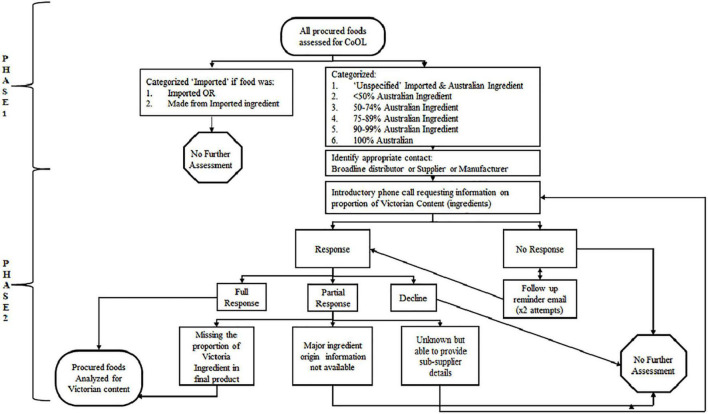
Participant recruitment process.

During Phase 1, all invoices were obtained across the menu cycle. These invoices identified all food items purchased for patient meals and the childcare centers, and the spend (including goods and service tax, GST) for each item. Product information data were identified for each food item including item name, purchase unit, item code, broadline distributor, manufacturer, supplier, country-of-origin-statement, and ingredient list. Food was then classified according to its Australian content: “Imported” included imported foods and foods made from entirely imported ingredient, “Unspecified” included foods with undefined amounts of imported and Australian ingredient, >50% Australian ingredient, 50–74% Australian ingredient, 75–89% Australian ingredient, 90–99% Australian ingredient and 100% Australian ingredient (Figure 1, Phase 1). Any food comprising 75% or more Australian ingredient was deemed to contain “significant” Australian content. Food items were also classified into one of the following food groups: “fruit, vegetables & water,” “grains & legumes,” “meats & alternatives,” “dairy & alternatives,” “fats & oils,” “discretionary,” “condiments & spreads,” and “ready-made meals.” Please refer to Table 1 below for a detailed description of each food group. Data were recorded in a spreadsheet in Microsoft Excel (Version 16.1, 2021).

**TABLE 1:** Food group category definitions.

Food group		Definition
1	Fruit & Vegetables & Water	Fresh and minimally processed fruit and vegetable items, juices, packaged water.
2	Grains & Legumes	Bread, rice, lentils, dried split peas, canned beans, flour, dry biscuits, etc.
3	Meats & Alternatives	Meat (beef, lamb & pork including mince, lean cuts & roasts), poultry (chicken & turkey including mince, lean cuts & roasts), fish fillets, tinned fish, eggs, tofu, etc.
4	Dairy & Alternatives	Milk, milk alternatives, flavored milks, yogurts, plain custard.
5	Fats & Oils	Oils, butter, margarine, cream, spray oils
6	Discretionary	High salt, fat and/or sugar products. As per Australian Dietary Guidelines (29), it includes processed meats (ham, bacon, sausages, chicken nuggets, frozen high fat burgers), desserts, pastries (e.g., croissant), muffins, hot chips
7	Spreads, Condiments & Spices	Sauces, spice mixes, salt, stocks, mayonnaise, vegemite, peanut butter, jams, honey
8	Ready Made Meals	Frozen meals (lasagne, veggie burgers), sandwiches, dried soup mix

During Phase 2, data from Phase 1 were used to identify all food items containing Australian grown content. These identified food items were then further assessed to determine their proportion of Victorian grown content. This information is not available on food labels, and therefore was requested from the various companies responsible for food production and procurements. All broadline distributors declined to participate, so suppliers and manufacturers were contacted and invited to contribute information about the proportion of Victorian grown ingredients in each food item (Figure 1, Phase 2). Contact details were obtained from hospital food procurement staff and manufacturer websites. If a supplier or manufacturer was unable to provide the requested information, they were asked to provide details for any relevant sub-suppliers. Downstream sub-suppliers within the supply chain were contacted and invited to participate. If an ingredient was known to be grown in Victoria but its contribution to the final product was unknown due to incomplete or partial responses, an estimation was made using its place on the ingredient list combined with known percentage contributions from other ingredients.

Data were collected by KF and two dietetic students.

### Data Analysis

Data were analyzed in Microsoft Excel to generate descriptive statistics (n, %) reporting on the country of origin and Victorian content by total food spend (expressed in Australian dollars). Data were presented for all items and according to food groups.

## Results

Total food spend over the 8-day audit period was $22,579 which comprised 252 food items from five broadline distributors and eight suppliers. Of the total food spend, “fruit, vegetables & water” accounted for the largest proportion (25%), followed by “discretionary foods” (16%), “dairy & alternatives” (15%), “grains & legumes” (14%), “meat & alternatives” (11%), “ready-made meals” (10%), “condiments, spreads & sauces” (7%), and “fats & oils” (2%).

### Food Products With Australian Grown Ingredient Content

**FIGURE 2:**
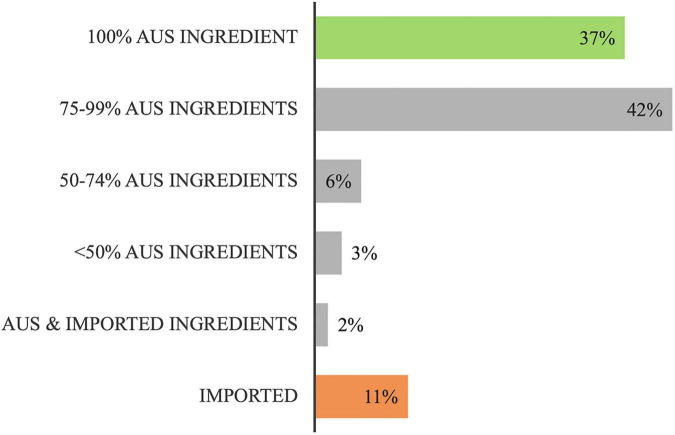
Distribution of the total food budget ($22,503.9) according to its food origins (Australian (Aus) or imported).

Overall, 37% of total food spend was on 100% Australian food items and 11% was spent on imported foods or foods with imported ingredients. The remainder, just over 50% of the total food budget, was spent on foods produced with some Australian grown ingredient content (Figure 2).

**FIGURE 3:**
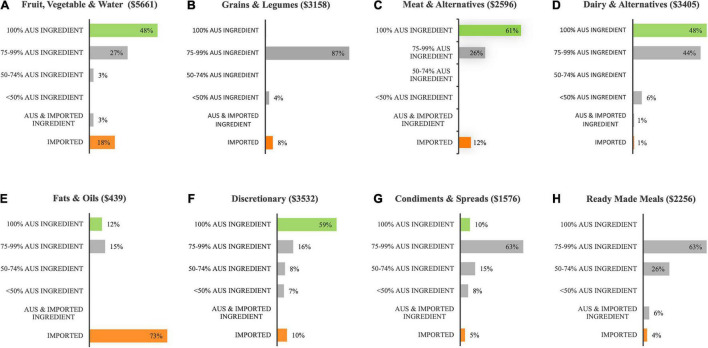
Distribution of the total food budget across eight food group categories **(A–H)**, according to its food origins [Australian (Aus) or imported].

Figure 3 shows more than half of the total spend for “meat & alternatives” (C) and “discretionary foods” (F), and almost half of the total spend for “fruit, vegetables & water” (A) and “dairy & alternatives” (D) was on 100% Australian product. These items comprised predominately fresh, unprocessed meats (including lean and minced cuts from beef, lamb & pork), poultry (including lean & minced cuts from chicken & whole turkey roast), eggs, fruit and vegetables, milk, portion control water, sugar, processed desserts, potato chips, and sweet biscuits. The “grains & legumes” (B), and “ready-made meals” (H) food groups included negligible 100% Australian product, but they both included high proportions (>60%) of foods containing significant Australian ingredient. These foods included breads, breakfast cereals, pasta and noodles, savory biscuits and sandwiches. The food group with the largest spend on imported product was “fats & oils” at 73% (E), but it must be noted that this food group contributed only 2% of the total food spend. Examples of food items that contained zero Australian content included cottonseed oil, coconut oil, fruit juices, frozen vegetables, canned tomato and apple, all canned legumes, rice and split green peas, fish and tofu, Asian sauces, dry gravy, seasoning, soup mixes, and two-minute noodles. Also of note, processed pork products (ham and bacon) from the discretionary group contained only minimal Australian ingredient.

### Food Products With Victorian Grown Ingredient Content

Of the 214 products that contained Australian grown content, 94 (44%) could not be assigned a proportion of Victorian grown content due to a lack of information. This was the result of manufacturer non-response, refusal to participate or a partial response (Table 2). Of the 46 items that received a partial response, only 18 had sufficient information to estimate their Victorian content. Full responses were received for 102 items, including 5 which were an acknowledged estimate by the manufacturer.

**TABLE 2:** Access to data from manufacturers on the Victorian content for each food item.

Response	Number	Proportion
No response	19	9%
Decline	4	2%
Decline + Explanation	43	20%
Partial response: 1 or more unknown ingredient origins	38*	18%
Partial response: proportion of Victorian ingredient not provided	8**	4%
Estimated response	5	2%
Full response	97	45%
Total	214	100%

*The proportion of Victorian grown ingredient could be estimated from 10 of these items. **The proportion of Victorian grown ingredient could be estimated from all these items.

**FIGURE 4:**
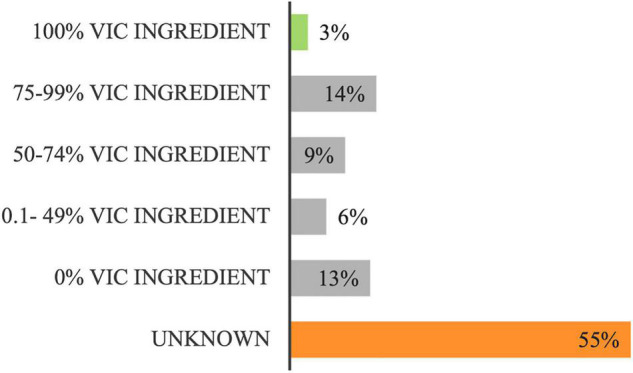
Distribution of the “Australian” food budget ($20,095) according to its proportion of Victorian (Vic) ingredient.

The proportion of the food budget spent on foods containing Australian ingredient over the 8-day audit cycle was $20,095 (89% of the total food budget). For 55% of the food budget spent on items containing Australian grown ingredient, the Victorian contribution was unknown (Figure 4), the reasons for which are described in Table 2. As described in Figure 4, 13% of foods containing Australian grown ingredient did not contain any Victorian content; just 3% was entirely grown in Victoria.

**FIGURE 5:**
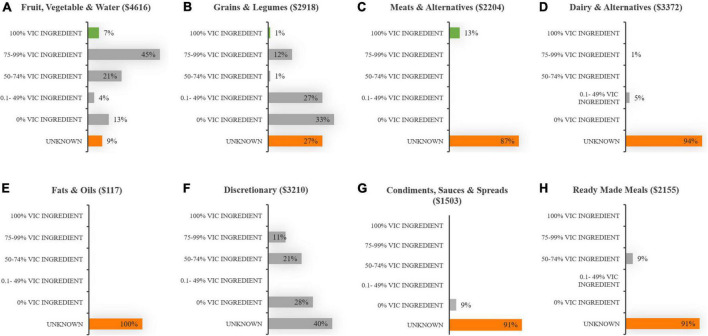
Distribution of the “Australian” food budget across eight food group categories **(A–H)**, according to its proportion of Victorian (Vic) ingredient.

Figure 5 highlights the difficulties in obtaining data on the proportion of Victorian ingredient for the following food groups: “fats & oils” (E), “condiments & spices” (G), “ready-made meals” (H), “dairy & alternatives” (D) and “meats & alternatives” (C). Seven percent of the “fruit, vegetable & water” food group budget was comprised of entirely Victorian grown food items. They included tinned tomatoes, fresh apples, bok choy, oregano, coriander & chives. Other food groups to include budget allocation for 100% Victorian products included “grains & legumes” (B) and “meat & alternatives” (C). Several Australian grown food items were identified as not having any Victorian content. These included tinned pineapple, banana, watermelon, sweet potato, portion-controlled water, gluten free grain products, biscuits, sugar, and several desserts with a high sugar and/or coconut content, salt and a number of portion-controlled sauces.

## Discussion

The purpose of this study was to describe, for the first time, the origins of food served in an Australian public hospital. With increasing interest in and commitment to a localized food system, measuring the proportion of a hospital’s food budget spent on foods with ingredient grown in Australia and, more specifically, within the state of Victoria provides a baseline upon which targets to increase local food purchase can be set, and changes over time monitored. While the audit revealed that only 37% of the hospital’s food budget was spent on entirely Australian grown food and drink, almost 80% of the food budget was spent on items containing significant (at least 75%) Australian content. Just 11% of the budget was spent on entirely imported food. Information from Australian manufacturers and suppliers concerning the Victorian content of their products was not forthcoming, with the requested data provided for less than half of the 214 food items purchased by the hospital. Consequently, the specific geographic origins of 55% of the budget spent on “Australian” food remains unknown.

The inability to access data from manufacturers on the origins of food grown in Australia was an unexpected yet key finding from this research. It is indicative of a complex and globalized food system where food can be sourced from a wide range of geographic regions, influenced by the seasons, climate events, import/export commodity prices and impacts due to COVID-19. This response was often given by large multinational companies who owned and operated multiple Australian processing plants, as well as suppliers of fresh, minimally processed perishable foods whose “holding” facilities were centralized near State borders. Both very large and very small companies cited intellectual property as a reason for declining to participate, while for products comprising multiple ingredients it tended to be harder to obtain a full response, in large part due to the need to contact sub-suppliers, who were also not forthcoming with information. Indeed, the more ingredients present in a food product, the more difficult it is to geographically define. It is interesting that the definition of “local” used by *Healthcare Without Harm* for multi-ingredient foods only require that 50% of ingredients, by weight, be grown and/or processed within a 240-mile radius of the healthcare facility (21).

In terms of establishing a local food procurement agenda, the lack of information relating to the origins of food supplied to healthcare is a fundamental challenge that will need to be overcome. Here, synergies with food safety programs can be harnessed for a dual purpose. Food safety programs in Australia are rigorous and standards are even higher in healthcare, where catering is provided to vulnerable and immunocompromised individuals (30). Indeed, all food “sold” in Australia should in theory be traceable to one step backward and one step forward at any point in the supply chain (31), and while recent incidents of microbial contamination within foreign foods have led to wider support for a more localized food system (32), it has also contributed to demand for enhanced traceability of food across the supply chain from “farm-to-fork” (32). This demand has seen a number of larger manufacturers employ crypto ledger technology which allows the transfer of efficient and transparent data up and down the processing chain (33) and in fact, a number of the larger manufacturers were willing to provide specific ingredient origin data but required a product batch number/identification-code to be able to deliver this information.

It is, however, important to recognize that these rigorous food safety requirements also present hurdles for smaller producers, who face increased expenses associated with delivering evidence of compliance (20, 21). An encouraging development though has seen the Australian Government working with Industry to develop a national approach to agriculture traceability systems (34).

Other opportunities for extracting geographic food origin information exist within institutional GPOs. These organizations, due to their scale, are able to nominate minimum “requirements” within catering agreements (20), which could include local ingredient content. But for this to be successful, the definition of “local food” would need to be addressed, including how to account for centralized processing systems, seasonal variation, and global prices. In these instances, when a seasonal menu change cannot be made, the yearly average “local” content would provide a more attainable and useful measure in deciding which products to preference.

Looking at the specific food items whose origin could be determined, this study, like other studies in the field (35, 36), reveals that the ability for the hospital to source local food reflects the agricultural activity in the region. A small proportion of items were identified as “Victorian grown and they included fresh apples, herbs, green leafy vegetables, red lentils, and poultry.” These items are supplied to the hospital from within Victoria year-round. Conversely, Australian food products that did not contain any Victorian content included sweet potato, banana, watermelon, tinned pineapple, sugar, portion control water and gluten free grain products. For most of these items there is no Victorian industry; they do not grow in colder climates. Indeed, an absence of Australian processing industry was identified for a number of imported products purchased for the hospital menu including cotton seed oil, coconut oil, fish, apple juice, canned legumes, frozen vegetables, tofu and Asian sauces. While some specialty products would not be expected to be made in Australia, it is surprising that domestically produced canned legumes, cottonseed oil, and fish are not available on the market, since Australia does produce these raw ingredients. Rather, these commodities are exported, or used for livestock feed in their raw (unprocessed) form (37–39). Australia also sources considerable amounts of frozen vegetables and fruit juice from overseas, despite producing adequate fruit and vegetables domestically. While there are a few Australian companies providing Australian grown alternatives, it is likely that the global commercial market for these products is too competitive. Certainly, the proportion of imported foods on the hospital menu is consistent with current Australian food import data (40). Although desirable, the reality is that an entirely “local” food supply may not be an achievable or a desired goal for a hospital menu.

Despite the lack of industry for certain food items, there is ample opportunity to expand the offering of Victorian seasonal fresh product on the menu. Melbourne’s food bowl (existing within 100 km of the city center) produces 47% of the vegetables and 8% the of fruit for Victoria as well as significant amounts of eggs and chicken meat (16), while the State of Victoria produces 40% of Australia’s meat (lamb & beef) and 63% of Australia’s dairy (by volume) (41). Considering this, it is disappointing that the Victorian content for fresh dairy, eggs and meat could not be ascertained. The findings above suggest that Victoria has the capacity to deliver local produce to hospitals provided there is support for appropriate procurement practices. Certainly, examples from abroad (20, 42) would indicate that, fresh produce provides the best avenue for establishing a local food to institution procurement initiative with inclusions for incremental expansion over time.

This study represents a snapshot of the origins of food purchased over 8-days by a hospital foodservice for patients, breast feeding mothers and 2 childcare centers. While the audit period reflects one full menu cycle, food items were not cross checked against the menu, so it is possible that data were not collected for all menu items. Furthermore, it was not possible to separate out food purchased for specific groups of consumers. While the findings provide insight into food supply at a metro Victorian public healthcare facility, they are unlikely to be reflective of other large Victorian metropolitan hospitals due to differences in meal production systems (e.g., cook fresh vs. cook chill) and menus (i.e., cycle vs. static menus), or regional Victorian hospitals who operate under different procurement policies. Similarly, more detailed analyses within each food group was not possible, although it is acknowledged that sub categories differ according to their nutrient yield, cost per nutrient yield and ability to be sourced from within Australia (e.g., muscle, offal, bone and insect meal within meat and alternatives).

This study found that a metro tertiary hospital in Victoria, adhering to current healthcare catering contracts, spent close to 80% of its food budget on foods with significant (>75%) Australian grown content, while just 11% was spent on entirely “imported” foods. This result was unsurprising and reflects current food import trends in Australia. Only 3% of the “Australian” food budget was spent entirely on Victorian grown ingredient, although this finding is likely an underestimate as the origins of 55% of this budget could not be ascertained. Seasons, climate events and global food markets are likely to influence the origins of ingredient both within processed and minimally processed “fresh” foods. The audit highlights the complexities of the globalized food system within which hospital food systems operate. Nonetheless, examples of farm to fork in healthcare from around the world show it is possible to disrupt the *status quo* to localize the food supply. Within Victoria, Australia, there is an opportunity for public healthcare to adopt a local food procurement strategy, based on government support, suitable growing conditions, and solid horticulture foundations. But certain requirements are needed to achieve this goal; transparent food origin information, a clear and measurable definition for local food, and an understanding of current Victorian food growing industries.

## Data Availability Statement

The datasets presented in this article are not readily available because informed consent to share data/datasets was not sought from or provided by the participants.

## Ethics Statement

The studies involving human participants were reviewed and approved by the Royal Children’s Hospital (Melbourne) Research Office. Participants provided verbal consent. Written informed consent for participation was not required for this study in accordance with the national legislation and the institutional requirements.

## Author Contributions

KF undertook data collection, analysis, and interpretation and drafted the manuscript. JC, JP, and HG supervised data collection and analyses and critically revised the manuscript. All authors contributed to design of the study and provided final approval of the version to be published.

## Conflict of Interest

KF and HG are employees at the health service where the research was undertaken. The remaining authors declare that the research was conducted in the absence of any commercial or financial relationships that could be construed as a potential conflict of interest.

## Publisher’s Note

All claims expressed in this article are solely those of the authors and do not necessarily represent those of their affiliated organizations, or those of the publisher, the editors and the reviewers. Any product that may be evaluated in this article, or claim that may be made by its manufacturer, is not guaranteed or endorsed by the publisher.
